# Acute, but not longer-term, exposure to environmental enrichment attenuates Pavlovian cue-evoked conditioned approach and Fos expression in the prefrontal cortex in mice

**DOI:** 10.1111/ejn.15146

**Published:** 2021-03-02

**Authors:** Gabriella Margetts-Smith, Anastasia I. Macnaghten, Leonie S. Brebner, Joseph J. Ziminski, Meike C. Sieburg, Jeffrey W. Grimm, Hans S. Crombag, Eisuke Koya

**Affiliations:** 1 Sussex Neuroscience, School of Psychology, University of Sussex, Falmer, UK; 2 University of Exeter College of Medicine and Health, Hatherly Laboratories, Exeter, UK; 3 Sussex Neuroscience, School of Life Sciences, University of Sussex, Falmer, UK; 4 Department of Neurochemistry, Graduate School of Medicine, The University of Tokyo, Tokyo, Japan; 5 Department of Psychology, University of Cambridge, Cambridge, UK; 6 Department of Biomedicine/DANDRITE, Aarhus University, Aarhus C, Denmark; 7 Department of Psychology and Program in Behavioral Neuroscience, Western Washington University, Bellingham, WA, USA

**Keywords:** cue reactivity, environmental enrichment, food seeking, Fos, Pavlovian appetitive conditioning, prefrontal cortex

## Abstract

Exposure to environmental enrichment can modify the impact of motivationally relevant stimuli. For instance, previous studies in rats have found that even a brief, acute (~1 day), but not chronic, exposure to environmentally enriched (EE) housing attenuates instrumental lever pressing for sucrose-associated cues in a conditioned reinforcement setup. Moreover, acute EE reduces corticoaccumbens activity, as measured by decreases in expression of the neuronal activity marker “Fos.” Currently, it is not known whether acute EE also reduces sucrose seeking and corticoaccumbens activity elicited by non-contingent or “forced” exposure to sucrose cues, which more closely resembles cue exposure encountered in daily life. We therefore measured the effects of acute/intermittent (1 day or 6 day of EE prior to test day) versus chronic (EE throughout conditioning lasting until test day) EE on the ability of a Pavlovian sucrose cue to elicit sucrose seeking (conditioned approach) and Fos expression in the medial prefrontal cortex (mPFC), orbitofrontal cortex (OFC), and nucleus accumbens (NAc) in mice. One day, but not 6 day or chronic EE, reduced sucrose seeking and Fos in the deep layers of the dorsal mPFC. By contrast, 1 day, 6 day, and chronic EE all reduced Fos in the shallow layers of the OFC. None of the EE manipulations modulated NAc Fos expression. We reveal how EE reduces behavioral reactivity to sucrose cues by reducing activity in select prefrontal cortical brain areas. Our work further demonstrates the robustness of EE in its ability to modulate various forms of reward-seeking across species.

## 1 | INTRODUCTION

The capacity to learn and remember associations between food rewards and the actions or cues that produce and/or predict their availability is essential for survival. In laboratory rats and mice, Pavlovian conditioning procedures demonstrate that following repeated presentations of a neutral stimulus (e.g. auditory cue) with a salient event such as food (US) delivery, the neutral stimulus acquires motivational significance and acts as a conditioned stimulus (CS) (Holland, 1993; Pavlov, 1927; Rescorla, 1988). Such CS’s are capable of eliciting approach responses towards food sources, serving as a conditioned reinforcers in their own right, and/or incentivize reward seeking behaviors (Cardinal et al., 2002; Fanselow & Wassum, 2015; Holland, 1977; Parkinson et al., 2000). Likewise, in humans, acquired incentive properties are apparent when a CS triggers conditioned emotional responses or increased food cravings that can motivate individuals to eat, and in some cases to overeat (Jansen, 1998; Jansen et al., 2011; Ridley-Siegert et al., 2015). Neuroscientific research over the years aimed at understanding the mechanisms by which CSs acquire and exert their incentive effects has identified brain areas such as the prefrontal cortex and nucleus accumbens as critical nodes in a wider forebrain network (Brebner et al., 2020; Cardinal et al., 2002; Day et al., 2006; Parkinson et al., 2000; Ziminski, Hessler, et al., 2017).

While much attention has been given to the psychological and neurobiological factors and mechanisms that promote CS-evoked reward seeking, much less is known about those that suppress these behaviors. Interestingly, in humans, cognitive and physical stimulation in the form of puzzle games or exercise reduces attentional bias towards food cues and food cravings (Oh & Taylor, 2013; Skorka-Brown et al., 2015). And while drawing parallels with studies In laboratory rodents is difficult, such stimulation may be provided through environmental enrichment (EE) procedures, where housing conditions include items such as toys, exercise wheels, and social enrichment, and cages are larger than standard laboratory housing (Mohammed et al., 2002; Nithianantharajah & Hannan, 2006; Solinas et al., 2020). Several studies by Grimm and colleagues demonstrate that even a brief, acute (~22 hr) exposure to EE attenuates cue-evoked sucrose seeking in a conditioned reinforcement task, indicated by reduced lever pressing for sucrose-associated cues (Grimm et al., 2008, 2013, 2016, 2019; Slaker et al., 2016; Glueck et al., 2017). Of course, in daily life many such food-associated cues are encountered passively and are forced onto us, e.g., in the form of televised, online, or print food advertisements. Whether and to what extent the incentive motivational properties of such passively experienced, Pavlovian conditioned cues are similarly modulated by acute EE exposure has not yet been tested.

Our aim here was therefore to examine the effects of acute and chronic EE housing conditions on cue-avoked sucrose seeking and neuronal activity in the corticoaccumbens network. To this end, we used a well-established appetitive Pavlovian conditioning procedure used previously in our lab, where mice learn to associate sucrose availability with presentation of an auditory CS. Following acquisition of this simple CS-US association, sucrose-seeking behavior is assessed by measuring approach and responding (i.e. head entry) to the sucrose delivery site during the (non-reinforced) sucrose-associated CS (Blaiss & Janak, 2009; Day et al., 2006; Sieburg et al., 2019; Ziminski, Hessler, et al., 2017). Next, because Fos expression increases in different corticoaccumbens areas in response to non-contingent food CS exposure (Brebner et al., 2020; Haight et al., 2017; Schroeder et al., 2001; Ziminski, Hessler, et al., 2017), we compared “Fos” expression across different EE conditions in the medial prefrontal cortex (mPFC), orbitofrontal cortex (OFC), and nucleus accumbens (NAc).

## 2 | MATERIALS AND METHODS

### 2.1 | Animals

C57BL/6J (wild-type) male mice were used in all experiments. Mice were either obtained from Charles River UK or bred at the University of Sussex. All mice were housed under a 12 hr light–dark cycle (lights on at 7:00 a.m.) at the maintained temperature of 21 ± 1°C and 50 ± 5% relative humidity. Mice were 9–10-week-age at the start of the experiments and were food restricted (90% baseline body weight) from 7 days before conditioning began until completion of the studies. All experiments were conducted in accordance with the UK 1986 Animal Scientific Procedures Act and received approval from the University of Sussex Animal Welfare and Ethics Review Board.

### 2.2 | Behavioral experiments

#### 2.2.1 | Apparatus

Similar apparatus and procedures were used as previously described (Ziminski, Hessler, et al., 2017). Briefly, behavioral training and testing were conducted in mouse-specific conditioning chambers (15.9 × 14 × 12.7 cm; Med Associates, Vermont, USA) each housed within a sound-attenuating and light-resistant cubicle. The chamber’s front and rear access panels and ceiling were constructed from clear Plexiglas, and the side walls were made from removable aluminum panels atop a stainless steel grid floor. A syringe pump dispensed 10% sucrose solution (serving as the US) into a recessed magazine receptacle fitted in the center of one of the side walls. This served as the unconditioned stimulus (US). The conditioned stimulus (CS) was an auditory click created by a mechanical relay. Experiment control and data collection was done using Med-PC IV (Med Associates).

#### 2.2.2 | Magazine training and Pavlovian conditioning

Mice first underwent a single magazine training session during which they received 40 ~15 μl sucrose solution deliveries, on a random interval-30 (RI-30) schedule. Next, mice underwent 12 conditioning sessions, 1–2 times daily over a 7-day period, in the morning (8:00 a.m. to 12:00 p.m.) and/or afternoon (12:00 p.m. to 4:00 p.m.). Each acquisition session lasted approximately 24 min and consisted of six 120 s CS presentations separated by RI-120s inter-trial interval (ITI) periods. During each CS period, ~15 μl deliveries of 10% sucrose solution were presented on a RI-30s schedule (i.e. on average 4 US deliveries per CS trial).

#### 2.2.3 | Behavioral testing

At 7–9 days following the last acquisition session, mice underwent a single test session for CS-elicited conditioned approach with the CS presented under the same schedule as conditioning, but in the absence of sucrose delivery (i.e. under extinction conditions). The number of head entries into the magazine during the CS and ITI were recorded.

#### 2.2.4 | Environmental enrichment

**FIGURE 1:**
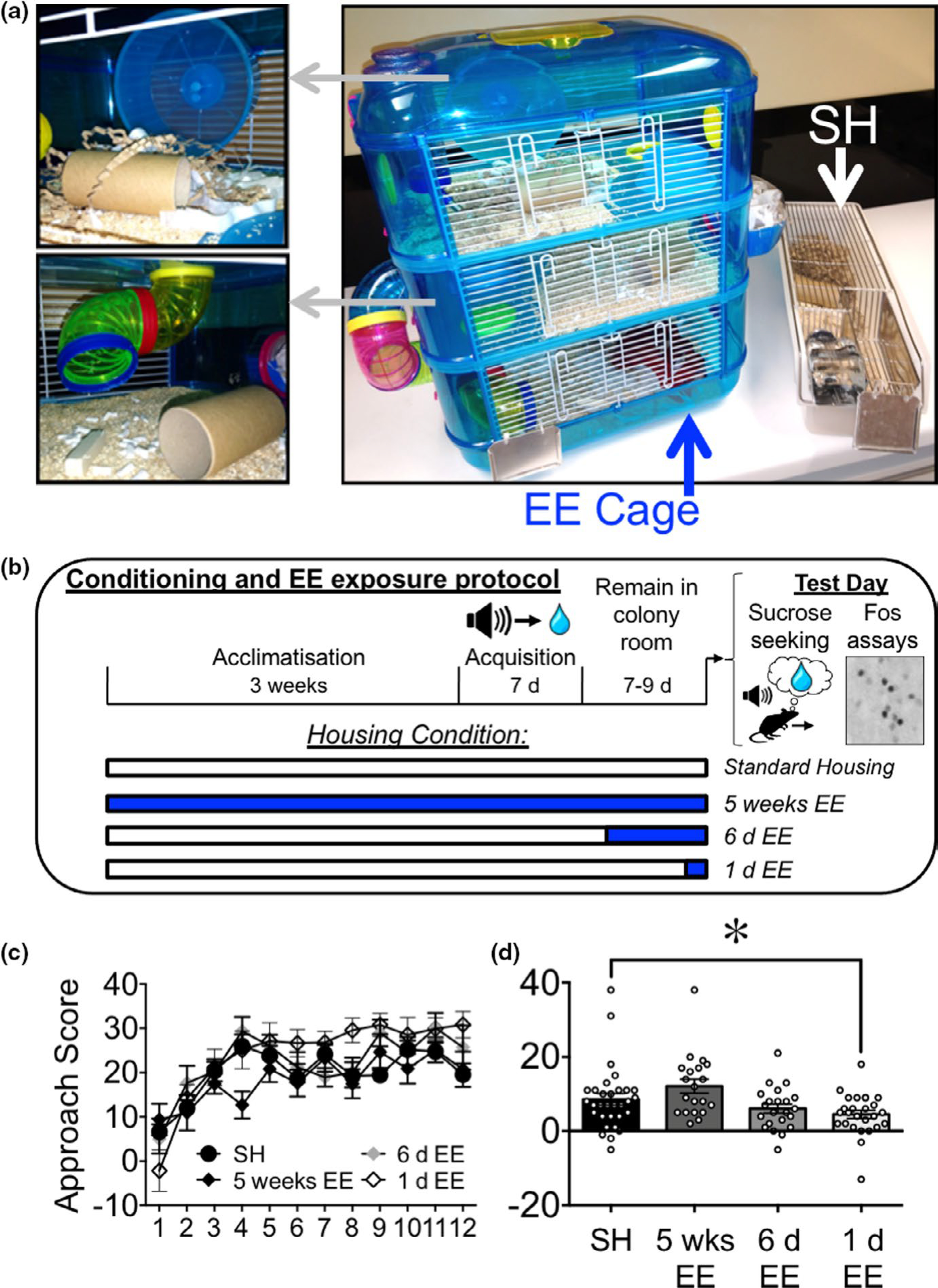
(a) The environmentally enriched (EE) housing cage and the standard housing (SH) cage. Gray dashed arrows indicate the inside of the EE cage. 1 day, but not 6 day and 5 weeks, of EE attenuates sucrose seeking elicited by Pavlovian sucrose cues. (b) Experimental timeline for 5 weeks EE (administered before and during acquisition until test day), the 1 day and 6 day EE (administered post-acquisition), and Standard Housing (SH) controls. (c) Approach Score as a function of the Acquisition session. (d) Approach Score on test day (*n* = 31, 20, 22, 26 for SH, 5 weeks, 6 day, and 1 day groups, respectively). *p < 0.05 against mice in the SH condition. All data are expressed as mean ± *SEM*

All mice were pair-housed and weaned into standard housing conditions; during the experiment mice were transferred to environmentally enriched housing at different time points (see Figure 1b). Standard housing consisted of a cage (48 × 15 × 13 cm) with basic nesting material and a wooden chew bar. Environmental enrichment (EE) housing consisted of 3 tiers (40 × 26 × 53 cm), with connecting tunnels, a separate sleeping pod, two exercise wheels, multiple forms of nesting material, a red plastic house, cardboard tunnels, and wooden chew bars (Figure 1a).

Four groups of mice consisting of three different EE exposure conditions and one standard housing (SH) control condition were trained and tested for CS-evoked Pavlovian approach (Figure 1d). In the 5 weeks (chronic) EE group (*n* = 20), EE was provided for 3 weeks prior to conditioning and continued during the 2 weeks of the behavioral experiments. By contrast, in the remaining two groups, EE was provided following acquisition of conditioning; either for 6 days (6 day EE; *n* = 22), or 1 day (1 day EE; *n* = 26) prior to testing. Mice in the standard-housed (SH; *n* = 31) control group remained in standard housing cages throughout the experiment.

### 2.3 | Fos immunohistochemistry

Ninety minutes following initiation of the final test session, mice were anaesthetized with 200 mg/kg sodium pentobarbital and transcardially perfused with phosphate-buffered saline (PBS; 137 mM NaCl, 10 mM PO_4_^3−^, 2.7 mM KCl, pH 7.4) and then 4% paraformaldehyde in PBS (PFA). Brains were post-fixated for 22 hr in 4% PFA, then cryoprotected with 30% sucrose in PBS before being frozen in dry ice and stored at −80°C. Coronal sections of 30 μm thickness containing the orbitofrontal cortex (OFC), medial prefrontal cortex (mPFC) and nucleus accumbens (NAc, AP 2.46, AP 1.94, and AP 1.18 respectively; (Paxinos & Franklin, 2001) were sliced on a Leica CM1900 cryostat and stored at 4°C in PBS-azide (PBS, 0.02% sodium azide).

Free-floating sections were washed in PBS three times for 10 min, before being incubated in PBS with 0.09% hydrogen peroxide for 20 min to quench endogenous peroxidase. Next, sections were washed three times in PBS, then blocked in PBST (PBS, 0.2% Triton X-100) with 3% normal goat serum (cat no. S-1000, RRID: AB_2336615; Vector Laboratories). The sections were then incubated in 1:800 anti-Fos primary antibody (cat no. 2250, RRID: AB_2247211; Cell Signaling Technology) in PBST with 3% normal goat serum at 4°C overnight.

The following day the slices were washed three times in PBS, then incubated for 2 hr in 1:600 biotinylated anti-rabbit secondary antibody (cat no. BA-1000, RRID: AB_2313606; Vector Laboratories) in PBST with 1% normal goat serum. Sections were washed three times in PBS before incubation with avidin–biotin complex (cat no. PK-4000, RRID: AB_2336818; Vector Laboratories) for 1 hr. Sections were washed two more times in PBS then incubated with 0.04% 3,3′-Diaminobenzidine-tetrahydrochloride (cat no. D5905; Sigma Aldrich) for ~2.5 min. After a final 2 washes in PBS, sections were mounted on Superfrost Plus slides (cat no. 10149870; Fisher) and left to dry overnight.

The next day, sections were serially dehydrated in graded ethanol baths and then cleared in Histo-Clear II (cat no. NAT1334; Scientific Laboratory Supplies) for 20 min. Slides were then sealed and coverslipped using HistoMount (cat no. NAT1308; Scientific Laboratory Supplies).

Representative images of the regions of interest (ROIs; Figure 3) were taken using a QI click camera (Qimaging) attached to an Olympus BX53 microscope running iVision software (version 4.0.15, RRID: SCR_014786; Biovision Technologies). Image analysis consisted of an automatic count of nuclei expressing high levels of Fos (Fos+) in predefined ROIs of 10X images using Fiji software (RRID: SCR_002285; NIH (Schindelin et al., 2012). During this count, images were submitted to a fast Fourier transform bandpass filter and inverted before being run through the 3D object counter plugin with a brightness threshold that depended on the average pixel brightness of the filtered image (Bolte & Cordelières, 2006).

### 2.4 | Data analysis

Cue-evoked behavioral responses were quantified by calculating an “Approach Score,” by subtracting head entries into the sucrose delivery magazine during CS trials from entries during the ITI periods. Approach scores during conditioning were analyzed with a two-way mixed ANOVA using the factors Housing Condition (Standard Housing, Chronic/5 weeks EE, 6 day EE, 1 day EE) and Session (1–12). Pavlovian Conditioned Approach Scores during the test session were analyzed using a one-way independent ANOVA comparing Conditioned Approach Scores with Housing Condition as a factor, followed by post-hoc analyses.

For the Fos expression analysis, 11–12 mice per group were randomly selected. Data from the behavioral and histological experiments were analyzed using Prism software (RRID:SCR_002798; GraphPad Software) and SPSS software (RRID:SCR_002865; IBM), and group data are presented as mean ± *SEM*.

Cell counts were analyzed using one-way ANOVA on the number of Fos+ cells per mm^2^ with Housing Condition as a factor. Analyses were conducted independently in the ventrolateral orbital frontal cortex (OFC), anterior cingulate cortex (ACC), prelimbic cortex (PL), infralimbic cortex (IL), and nucleus accumbens core (NAc^Core^) and shell (NAc^Shell^). Further independent ANOVAs were conducted for the laminar analyses in the OFC and mPFC areas separating shallow (II-III) and deep (V-VI) layers. Layers were defined using criteria described in Van De Werd et al., (2010). All *post hoc* analyses were conducted using Fisher’s LSD multiple comparisons, comparing each EE condition to the standard-housed (SH) control.

**FIGURE 2:**
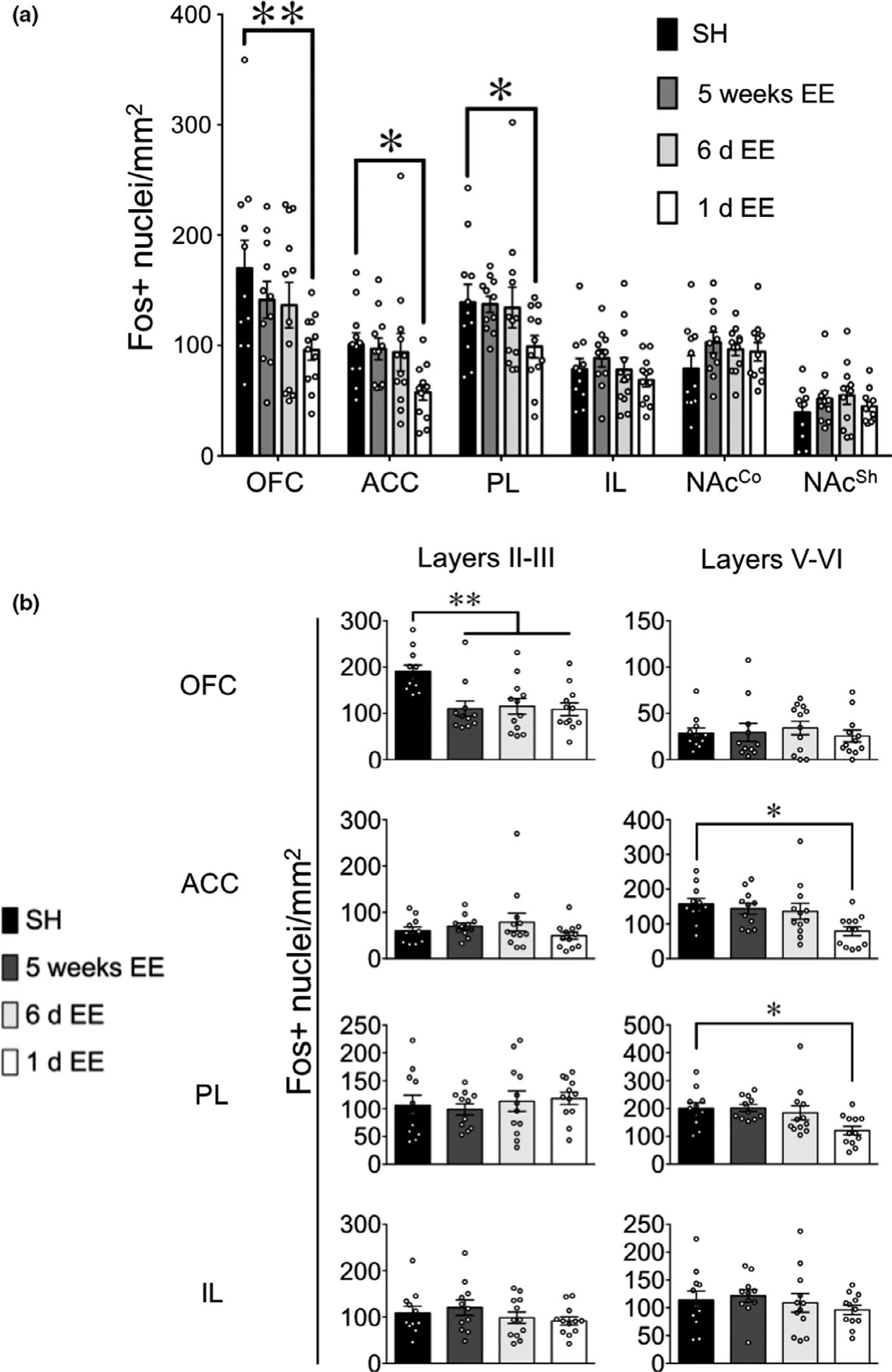
EE differentially modulates Fos expression in the prefrontal cortex, but not nucleus accumbens (NAc), subareas following testing for sucrose seeking (*n* = 11–12 per group). (a) Fos expression in the prefrontal cortex and NAc. (b) Laminar-based analyses of Fos expression in prefrontal cortex subareas. *Legend*: OFC, orbitofrontal cortex; ACC, anterior cingulate cortex; PL, prelimbic cortex; IL, infralimbic cortex; NAc^Co^ and NAc^Sh^, nucleus accumbens core and shell, respectively. *p < 0.05, **<0.01, compared with mice in SH condition. All data are expressed as mean ± *SEM*

In addition, we performed estimation statistics on Test Day data for the Approach Score (Figure 1d) and Fos counts (Figure 2a,b) using the Shared Control Estimation Plot function on https://www.estimationstats.com/#/. This method uses 5,000 bootstrap samples to calculate the lower and upper bounds of the 95% confidence interval (CI), see Supporting Materials Figures S1–S3 for plots with CIs). The effect size (i.e. mean differences between experimental group (5 weeks EE, 6 day EE, or 1 day EE) – SH control group) and CIs are reported as *effect size [CI width lower bound; upper bound*]*)*. This approach provides additional information regarding the confidence and likelihood of the effect size (Calin-Jageman & Cumming, 2019; Ho et al., 2019).

## 3 | RESULTS

### 3.1 | The effects of EE on sucrose seeking induced by Pavlovian sucrose cues

Four groups of mice were trained on a Pavlovian sucrose conditioning task (Figure 1b) for them to acquire an association between sucrose reward and a cue that predicts its availability. All mice in this task received auditory cue (CS) presentations explicitly paired with 10% sucrose solution (US) during each acquisition session (Figure 1c) for a total of 12 sessions. There was a significant effect of Session on the Approach Score (*F*_11,1045_ = 19.17, *p*< 0.001) indicating that there was an increase in overall approach scores as the sessions progressed (Figure 1c). This suggests that the mice reliably acquired the CS–US association during training. Whilst there was a significant interaction between Housing Condition and Session (*F*_33,1045_ = 1.66, *p* < 0.05), there was no main effect of Condition on the Approach Score (*F*_3,95_ = 2.18, *p* < 0.09). Due to this interaction, we further analyzed the final three sessions of training where the Approach Score appeared to asymptote. There was no significant interaction between Housing Condition and Session (*F*_6,190_ = 0.92, *p* = 0.48), nor main effects of Housing Condition (*F*_3,95_ = 1.87, *p* = 0.14) and Session (*F*_2,190_ = 2.14, *p* = 0.12). Taken together, the significant interaction during conditioning reflected small (but significant) differences in the rate of acquisition of the conditioned response. However, with sufficient training, behavioral performance toward the end of the acquisition phase (i.e., prior to testing) was stable and equal for the conditions.

Seven to nine days following the last acquisition session, on the test day there was a significant effect of Housing Condition on the Conditioned Approach Score (*F*_3,95_ = 4.64, *p* < 0.01). Subsequent post hoc analyses comparing each EE condition to the standard housing (SH) control showed a significant decrease in conditioned approach following 1 day EE; *p* < 0.05, −4.1; [−8.4, 0.8]; Figure 1d). Compared with SH, there was no effect on the Approach Score following 5-week or 6-day EE (3.5; [−0.9, 8.6]; *p* = 0.10 and −2.5 [−6.7, 0.93] *p* = 0.21 respectively). Thus, cue-evoked conditioned approach response was reduced only when EE was experienced following conditioning and 1 day prior to testing.

#### 3.1.1 | Cue-evoked Fos expression

**FIGURE 3:**
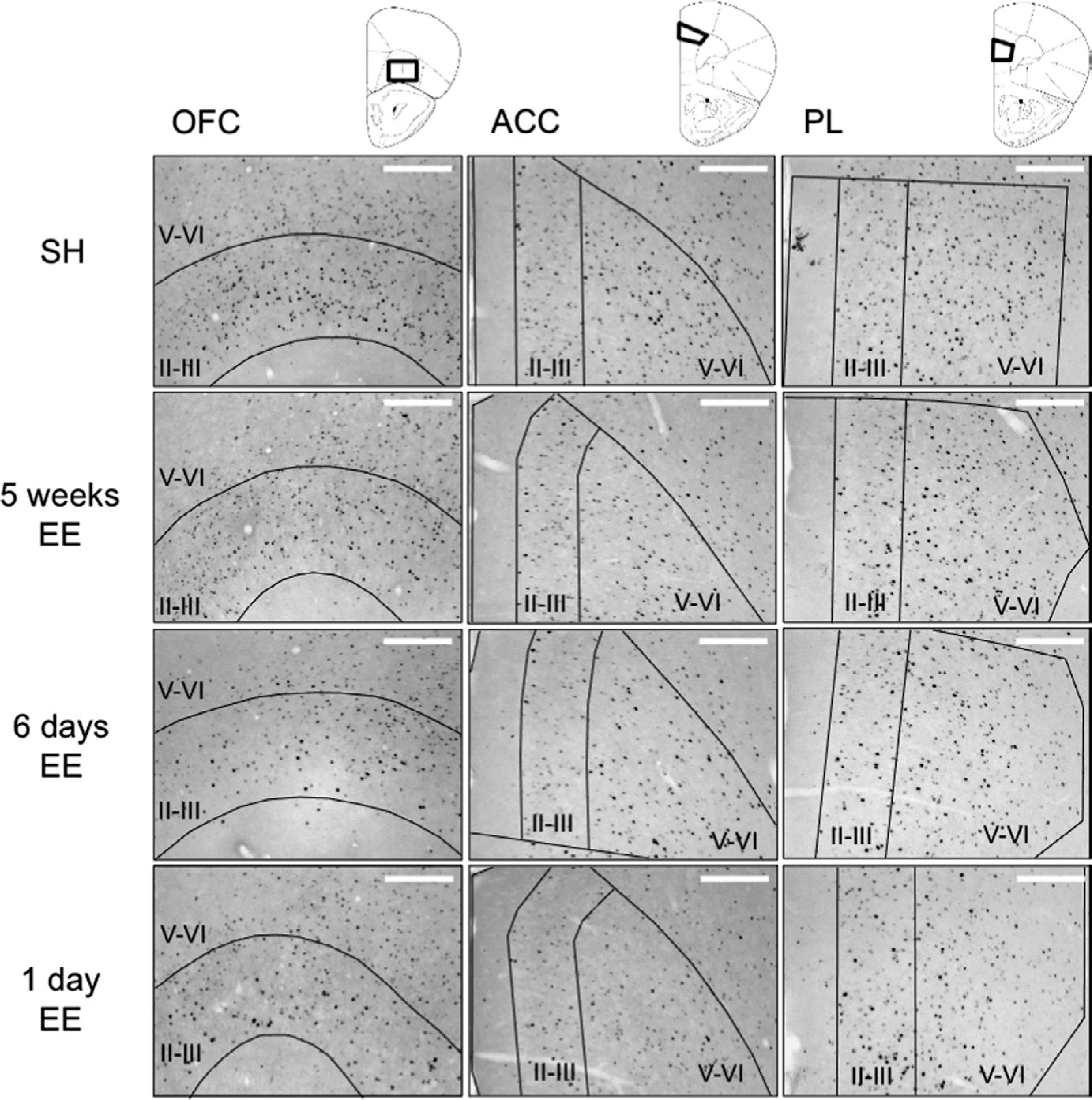
Representations of coronal sections indicating regions used for Fos expression analyses in prefrontal cortex subareas adapted from Paxinos and Franklin (2001) (top panel). Representative images of Fos expression in prefrontal cortex subareas (bottom panel; white scale bar = 200 μm). *Legend*: II-III, shallow layers II-III; V-VI, deep layers V-VI

Following the test session for Pavlovian conditioned approach, we performed immunohistochemistry for the neuronal activity marker “Fos” (Cruz et al., 2013) to examine EE-modulated changes in neuronal activity in subregions of the prefrontal cortex and nucleus accumbens (Figures 2 and 3). There was a significant effect of Housing Condition on Fos expression in the anterior cingulate cortex (ACC, *F*_3,42_ = 2.90, *p* < 0.05). Post hoc analyses indicated that Fos expression decreased in the 1-day EE condition (−43.4; [−68.5, −20.7]; *p* < 0.05). However, and in line with the behavioral results on test, 5-week and 6-day EE had no effect on Fos expression (−4.2; [−31.2, 23.5], *p* = 0.81; and −7.3; [−38.4, 41.3], *p* = 0.67, respectively; Figure 2a). No significant effects of Housing Condition were seen in the orbitofrontal cortex (OFC, *p* = 0.06; −28.5 [−91.9, 21.4] for 5-week EE; −33.4 [−102.5, 21.7] for 6-day EE; −73.9 [−135, −30.3] for 1-day EE), prelimbic Cortex (PL, *p* = 0.13; −1.8 [−37.4, 28.5] for 5-week EE; −5.0 [−46, 45.2] for 6-day EE; −40.4 [−79.0, −7.5] for 1-day EE), infralimbic cortex (IL, *p* = 0.47; 10.1 [−17.7, 30.8] for 5-week EE; −0.37 [−27.2, 26.7] for 6-day EE; −9.7 [−35.6, 8.8]), nucleus accumbens shell (NAc^Shell^, *p* = 0.31; 12.4 [−4.6, 33.5] for 5-week EE; 15.5 [−3.7, 37.7] for 6-day EE; 5.5 [−9.06, 22.4] for 1-day EE) or core (NAc^Core^, *p* = 0.40; 23.7 [−6.6, 50.5] for 5-week EE; 17.4 [−10.4, 39.4] for 6-day EE; 15.2 [−13.7, 41.0] for 1-day EE). Overall, these data suggest that 1 day of exposure to enriched housing following conditioning, but not the more prolonged 6 days or 5 weeks, attenuated both the behavioral response and Fos expression in the ACC following sucrose cue exposure.

Because of the robust effects of EE on reductions in Fos in the ACC, as well as decreasing trends in the OFC and PL, a more in-depth laminar analysis was conducted (Figures 2b and 3). The OFC, ACC, PL, and IL were divided into shallow (layers II-III) and deep (layers V-VI) areas with distinct chemo- and cyto-architectural features and connectivity (Van Riga et al., 2014; De Werd et al., 2010). There was a significant effect of Housing Condition on Fos expression in the OFC shallow layers (*F*_3,42_ = 6.60, *p* < 0.001) and the ACC and PL deep layers (*F*_3,42_ = 4.02, *p* < 0.05; *F*_3,42_ = 4.02, *p* < 0.05). Post hoc analyses showed that in the OFC, significant decreases in Fos expression occurred in all EE conditions as compared with SH controls; *p* < 0.01, −80.7 [−115, −33.6] for 5-week EE; *p* < 0.01 −75.5 [−115.0, −35.6] for 6-day EE; *p* < 0.01, −81.9 [−117.5, −45.9] for 1-day EE. By contrast, decreases in Fos expression were only observed in the 1-day EE group in the ACC and PL deep layers (ACC: *p* = 0.59, −13.5 [−56.4, 29.8] for 5-week EE; *p* = 0.40, −20.9 [−66.4, 42.7] for 6-day EE; *p* < 0.01, −78.5 [−116, −39.0] for 1-day EE; PL: *p* = 0.96, 1.39 [−44.8, 43.6] for 5-weeks EE; *p* = 0.56, −16.1 [−67.8, 57.1] for 6-day EE; *p* < 0.01, −79.9 [−129.7, −32.2] for 1-day EE).

By contrast, no significant effects of Housing Condition on Fos were observed for the IL (shallow layers: *F*_3,42_ = 0.91, *p* = 0.45, 11.3 [−30.2, 52.0] for 5-week EE, −10.5 [−50.6, 21.8] for 6-day EE, −17.5 [−54.2, 10.5]; and deep layers: *F*_3,42_ = 0.61, *p* = 0.61, mean difference 7.7 [−33.4, 42.9] for 5 weeks EE, −5.0 [−48.2, 40.2] for 6 day EE, −17.7 [−57.0, 13.9] for 1 day EE ), and OFC deep layers (*F*_3,42_ = 0.26, *p* = 0.86, 0.8 [−16.6, 27.4] for 5-week EE, 5.6 [−14.7, 20.9] for 6-day EE, −3.3 [−19.5, 12.6] for 1 day EE), ACC shallow layers (*F*_3,42_ = 1.11, *p* = 0.35, 9.9 [−10.9, 28.8] for 5-week EE, 18.7 [−9.4, 80.8] for 6-day EE, −10.4 [−30.8, 10.6] for 1-day EE ), and PL shallow layers (*F*_3,42_ = 0.33, *p* = 0.80, mean difference −7.4 [−48.9, 28.4] for 5-week EE; 7.3 [−41.8, 56.7] for 6-day EE; 12.3 [−32.0, 47.9] for 1-day EE).

## 4 | DISCUSSION

A number of studies have revealed how acute EE exposure attenuates cue-dependent sucrose seeking (conditioned reinforcement) in rats (Grimm et al., 2008, 2013, 2016; Slaker et al., 2016). Here we examined the effects of EE exposure on the ability of a non-response contingent cue to elicit sucrose seeking in the form of conditioned approach responding in mice. We found that 1-day, but not 6 day or chronic (5 weeks), EE exposure attenuated cue-evoked sucrose seeking. In parallel, we saw decreases in neuronal activity in certain subareas of the prefrontal cortex in the 1-day group, but we did not observe any effects of EE on Fos in the NAc. More specifically, in the ACC and PL, 1 day (but not 6 day or 5-week EE) reduced Fos expression in the deep, but not shallow layers of the dorsal mPFC (dmPFC; ACC and PL). By contrast, in the OFC, all EE exposure conditions attenuated Fos in the shallow, but not deep layers. With these results, we shed new light on the potential prefrontal cortical mechanisms of how acute EE exerts its effects on motivated actions that are controlled (or at least elicited) by Pavlovian cues. Our findings, together with previous studies, highlight EE’s robust ability to impact across different motivational qualities of incentive cues (lever pressing for sucrose cues versus cue-evoked approach behavior) in different species (rats versus mice).

### 4.1 | Potential psychological mechanisms of acute EE effects

Changes in the perception of environmental stimuli can be evaluated in relation to prior experiences with other environmental stimuli. For instance, returning to work may feel rather mundane immediately following an exciting holiday. This type of “contrast effect” (Black, 1968; Flaherty, 1982) may provide a candidate mechanism for how acute EE diminished the impact of the sucrose-associated cue, and we can speculate about a number of ways this may have worked.

One possibility is that the contrast effect may arise from evaluations that are made between EE versus the test environment. In contrast to standard housing, EE allows increased opportunities to engage in naturalistic behaviors that satisfies the basic behavioral needs of animals, such as foraging and exploration (Nithianantharajah & Hannan, 2006). When placed in the test context, EE’s novel and stimulating experience may have rendered these mice to pay less attention toward familiar sucrose-associated cues and physical features of the test chamber, thus attenuating sucrose seeking.

Additionally, reductions in sucrose seeking may have been the result of changes in the perceived value of sucrose reward because of a direct contrast with EE experience. Indeed, based on the evidence of conditioned anticipatory responses that rats exhibit before entering EE housing (van der Harst et al., 2003), we might consider that EE experience can itself be rewarding in some manner. One caveat here is that it is difficult to directly compare the rewarding value of sucrose (an ingestive reward) against EE (reward gained through exploratory experience) because they differ in in many ways, including their sensory modalities and time course. That said, the reduction in cue-evoked sucrose seeking is reminiscent of the “successive negative contrast effect” in which a behavioral response to a reward is attenuated due to experience with a larger reward (Black, 1968; Flaherty, 1982). On test day, our mice did not directly experience sucrose reward. However, we and others have shown that conditioned approach responses evoked by Pavlovian sucrose cues are under the control of retrieving a representation of the sucrose reward, as it is sensitive to devaluation manipulations (Sieburg et al., 2019). From this perspective, our mice may have attributed greater reward value to the recent EE experience compared with the retrieved representation of sucrose, thus resulting in a negative contrast effect (Grimm & Sauter, 2020).

Somewhat consistent with these notions is that, in contrast to 1 day EE experience, 6 day EE or more chronic EE exposure did not attenuate sucrose seeking. These data indicate that in the 6 day and chronic EE conditions, the reductions in EE’s novelty as a result of prolonged exposure had modulated the contrast effects. When the test context is compared with a less novel and stimulating EE condition, mice may have paid close attention to environmental stimuli in the test context, and thus exhibited sucrose seeking. Alternatively, because novelty itself has rewarding properties (Jaegle et al., 2019), a possibility here is that the decreased novelty of EE resulted in its diminished reward value, and therefore reduced contrast.

### 4.2 | The implications of reduced activity in prefrontal cortex areas following acute EE

Our observed reductions in OFC and dmPFC Fos expression following acute 1 day EE exposure are consistent with a recent study which reported similar acute EE-mediated Fos reductions under conditioned reinforcement conditions (Grimm et al., 2016). As the inactivation of the OFC and dmPFC result in a reduction of various forms of cue-evoked reward seeking behaviors (Calu et al., 2013; Fuchs et al., 2004), our observed Fos reductions may indicate reduced motivation to seek sucrose. In the OFC, Fos reductions may reflect attenuation of the motivational qualities of the cue itself. In support of this idea, a previous study by Flagel et al found that cue-evoked OFC *Fos* mRNA expression is associated with cue-controlled sign tracking, when cues themselves become sought after (Flagel et al., 2011). Additionally, we have observed reductions in OFC *Fos* expression following extinction of cue-evoked conditioned approach in sucrose conditioned mice, which may reflect reduced salience (or attention) to reward-associated cues (Ziminski, Hessler, et al., 2017).

Our observed reductions in OFC and dmPFC Fos levels may provide clues about alterations in the wider motivational network in which the PFC serves as a critical node (Gourley & Taylor, 2016; Kalivas et al., 2005). First, these areas receive reciprocal excitatory connections with the basolateral nucleus of the amygdala (BLA) (Hoover & Vertes, 2007; Mcdonald et al., 1996). This area is necessary for guiding flexible behavioral responses that are dependent on retrieving a representation of a learned rewarding outcome, because lesions and inactivation of this area render animals insensitive to reward devaluation (Lichtenberg et al., 2017; Pickens et al., 2003; Wassum & Izquierdo, 2015). Therefore, reduced OFC and dmPFC Fos expression may result from reduced BLA activity, which may signal contrasts in reward magnitude that result in decreased sucrose seeking.

We observed reductions in Fos from the deep layers of the mPFC, which receives considerably more dopaminergic input from the ventral tegmental area than the shallow layers (Van Eden et al., 1987). Fos expression of this area during food seeking is dependent on dopamine 1-receptor (D1-R) activation, as systemic D1-R antagonism attenuates this behavior, as well as Fos in the dmPFC (Nair et al., 2011). As such, our Fos reductions here may be indicative of reduced D1-R signaling. In support of this idea, D1-R agonism reverses the EE-mediated attenuation of cue-evoked sucrose seeking in operant-conditioned rats (Glueck et al., 2017). Finally, the deep layer neurons of the mPFC neurons project to motivationally-relevant subcortical structures such as the nucleus accumbens and PVT (Berendse et al., 1992; Gabbott et al., 2005; Otis et al., 2013). Thus, this Fos reduction may reflect reduced activity to these areas, which may then attenuate sucrose seeking.

In contrast to the mPFC, reductions in Fos were observed in the shallow layers in the OFC. The shallow layers contain a significant proportion of intra-cortical excitatory projection neurons (Douglas & Martin, 2004), and the OFC sends projections to the dmPFC (Bedwell et al., 2014; Hoover & Vertes, 2007). Given this connection, it is tempting to speculate that reduction in the activity of these shallow layer OFC neurons coordinates the dampening of sucrose seeking by reducing activity in the dmPFC. Thus, one interesting line of future investigation would be to selectively stimulate the activity of this OFC to dmPFC projection using chemo/optogenetic approaches and determine if this would be sufficient to override acute EE effects.

We have recently observed reductions in NAc *Fos* mRNA and Fos expression following extinction of conditioned approach and devaluation of sucrose reward, respectively (Sieburg et al., 2019; Ziminski, Hessler, et al., 2017). Hence, it was surprising that we did not detect any reductions in Fos in this structure. However, we and others have observed that different sets of cues recruit neurons with opposing behavioral responses or neurophysiological features in the absence of changes in Fos expression (Suto et al., 2016; Ziminski et al., 2017). As such, EE may exert its effects via a different NAc neuronal mechanism compared with extinction and devaluation, i.e. selecting a new group of neurons without any changes in the number of activated neurons. Therefore, future studies need to determine this possibility using tools, such as the *TetTag H2BGFP* mouse, that label different groups of cue-activated neurons at different time points, i.e. before and after EE exposure (Tayler et al., 2013). Finally, Fos reductions in the NAc following acute EE have been reported in operant-conditioned rats that exhibited attenuated lever pressing for sucrose cues (Grimm et al., 2016). These differences may reflect the different neuronal substrates that subserve the conditioned reinforcing properties of the appetitive cues versus conditioned approach behaviors (Parkinson et al., 2000; Wassum et al., 2011). Also, unlike the previous study by Grimm et al., there was no difference in social enrichment between the EE and standard housing conditions. Therefore, this more pronounced difference in housing condition may have contributed to more robust differences in reductions in NAc Fos in their study.

### 4.3 | The implications of reduced OFC activity across all EE conditions

In the OFC, reduced Fos was observed across all EE conditions. As the 6 day and chronic EE conditions did not exhibit alterations in conditioned approach compared with controls, this prolonged (days, weeks) exposure to EE produces neuronal adaptations independently from changes in motivation and/or reward value. Our findings here suggest that simply reducing activity in the OFC shallow layers is not sufficient to reduce sucrose seeking and highlight how Fos levels are not necessarily influenced by changes in behavioral output on test day. Mounting evidence from our group and others demonstrate that distinct, sparse sets of activated neurons or “neuronal ensembles” in the prefrontal cortex mediate cue-evoked reward seeking for food and drug rewards (Laque et al., 2019; Suto et al., 2016; Warren et al., 2016, 2019; Whitaker et al., 2017). These findings raise the possibility that while long exposure to EE may reduce activity in the OFC more generally, it may not necessarily do so in neuronal ensembles which subserves conditioned approach responses. To confirm this idea, further studies utilizing approaches, such as the *TetTag H2BGFP* mice (Tayler et al., 2013), that allow tagging of cue-activated neurons and then monitor their reduction in activity following prolonged EE exposure need to be performed.

Finally, one caveat of this study is that we examined Fos expression following the expression of sucrose seeking. Hence, we do not know whether EE exposure itself modulated Fos expression prior to testing due to exposure to a novel environment. However, this possibility may not be likely because dmPFC Fos expression peaks at approximately 1.5–2 hr following a single exposure to a novel environment and stress exposure and returns to baseline in 18–24 hr (Brebner et al., 2020; Cifani et al., 2012). Moreover, dmPFC Fos expression habituates in response to repeated exposure to a novel environment and returning to baseline levels (Struthers et al., 2005). In both cases, Fos expression returns to baseline, but does not decrease below these values. Another caveat here is that we only used male mice. Indeed in humans, women have reported to experience more cravings for sweet foods (e.g. chocolate) compared with men (Zellner et al., 1999). Of relevance to this study, female rats displayed more pronounced cue-evoked approach behavior during a sucrose conditioning task and under extinction conditions, indicating sex differences in the learning of food-cue associations and/or the motivational impact of such cues (Hammerslag & Gulley, 2014). Therefore, it is important in future studies to address whether there are differences in EE’s ability to modulate neurobehavioral responses to food cues between male and female animals, to determine how generalizable EE’s efficacy is.

## 5 | CONCLUSIONS AND FUTURE DIRECTIONS

We show that brief EE exposure powerfully reduces reward seeking induced by non-contingent exposure to Pavlovian cues in mice by attenuating activity in the shallow and deep layers of the OFC and dmPFC respectively. Our study reinforces the effectiveness of EE as a non-pharmacological intervention that confers resilience against various forms of reactivity to food cues across species. In future studies, it would be important to determine which cortical (e.g. other PFC areas) and subcortical brain areas (e.g. NAc, amygdala) these Fos-expressing neurons project to using retrograde tracing approaches. Following such identification, the causal role of these pathways in EE’s suppressive effects can be determined using chemo/optogenetic strategies. Doing so will obtain a more comprehensive picture of the wider PFC network that contributes to the reduced drive to seek sucrose. As food cue exposure can be a potent trigger for conditioned food cravings and eating (Jansen, 1998; Jansen et al., 2011; Ridley-Siegert et al., 2015), identifying this network will provide the much needed insight into how the brain can harness its anti-craving mechanisms and better control excessive forms of eating.

## Supplementary Material

supportingfigures

## Abbreviations:

ACC: Anterior cingulate cortex
AP: Anterior posterior
CAS: Conditioned approach score
CS: Conditioned stimulus
dmPFC: Dorsal medial prefrontal cortex
EE: Environmental enrichment
IL: Infralimbic cortex
ITI: Inter-trial interval
mPFC: Medial prefrontal cortex
NAc: Nucleus accumbens
OFC: Orbitofrontal cortex
PBS: Phosphate buffered saline
PFA: Paraformaldehyde
PL: Prelimbic cortex
ROI: Region of interest
SEM: Standard error of the mean
SH: Standard housing
US: Unconditioned stimulus
