# Supplementary material for: Acute, but not longer-term, exposure to environmental enrichment attenuates Pavlovian cue-evoked conditioned approach and Fos expression in the prefrontal cortex in mice
Source: Eur J Neurosci. Author manuscript; Available in PMC 2022 Apr 1. (PMC8085094; doi:10.1111/ejn.15146)
Supplement: supportingfigures [file NIHMS1686117-supplement-supportingfigures.pdf]

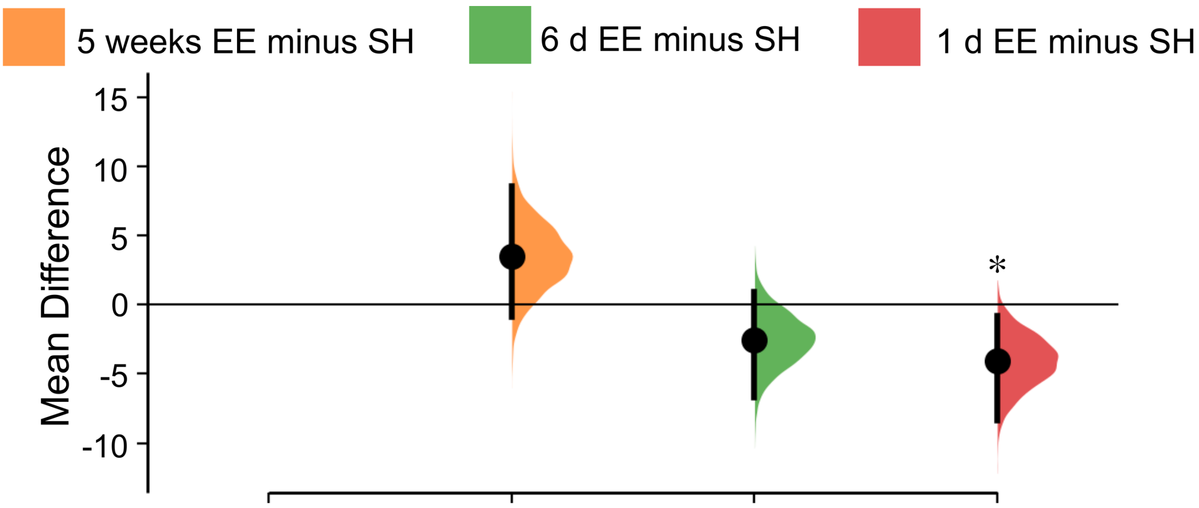

Supporting Figure 1

The mean difference for 3 comparisons of Approach Scores against the shared Standard Housing (SH) condition are shown in the above plot where mean differences are plotted as bootstrap sampling distributions. Each mean difference is depicted as a dot. Each 95% confidence interval is indicated by the ends of the vertical error bars. *$p<0.05$ compared to SH.

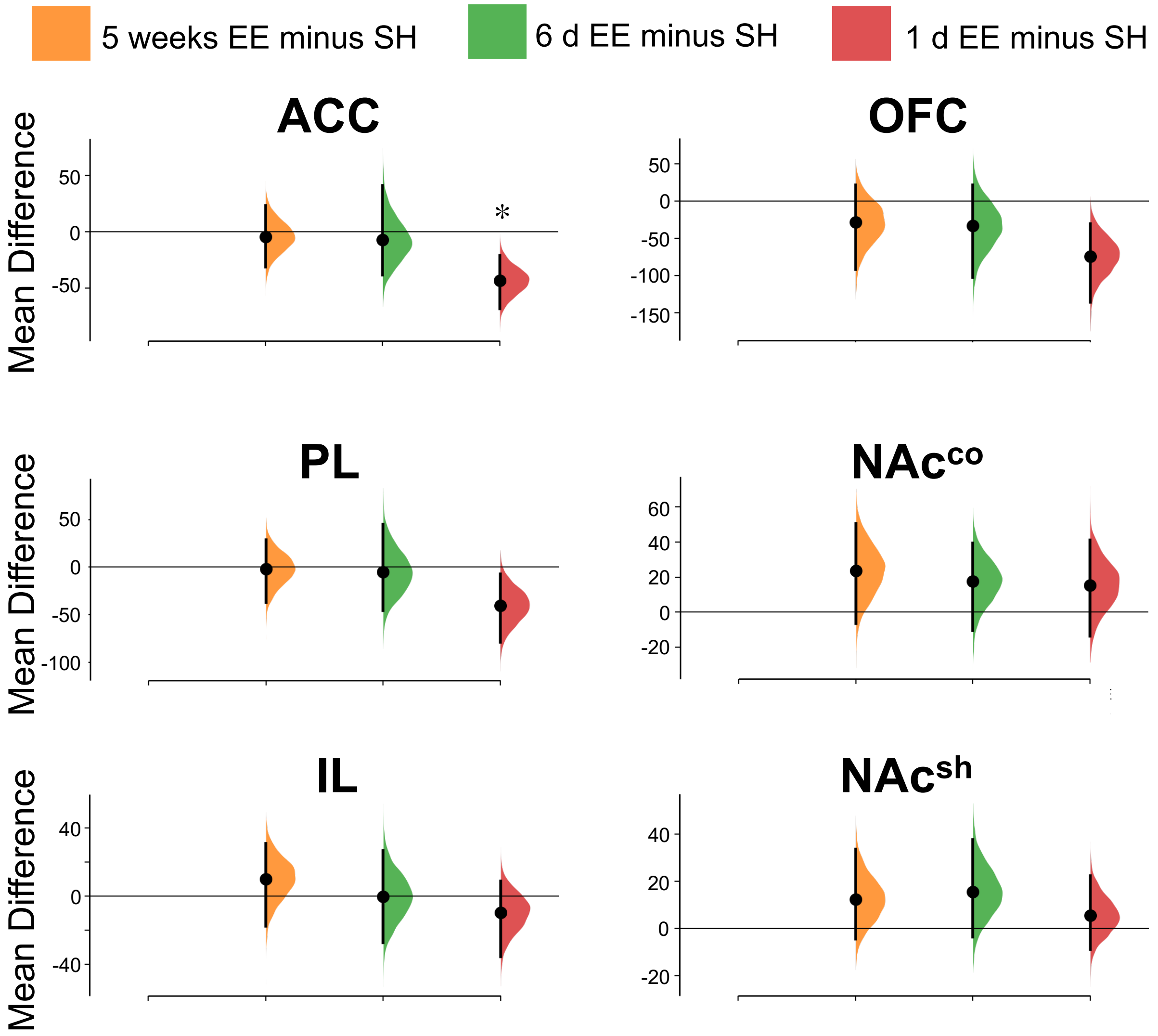

Supporting Figure 2

Fos expression analyses in prefrontal cortex and nucleus accumbens subareas. The mean difference for 3 comparisons of Fos counts (cells/mm^2) against the shared Standard Housing (SH) condition are shown in the above plots where mean differences are plotted as bootstrap sampling distributions. Each mean difference is depicted as a dot. Each 95% confidence interval is indicated by the ends of the vertical error bars. *$p<0.05$ compared to SH. *Legend*: OFC=orbitofrontal cortex, ACC=anterior cingulate cortex, PL=prelimbic cortex, IL=infralimbic cortex, NAc^{Co} and NAc^{Sh}=nucleus accumbens core and shell, respectively.

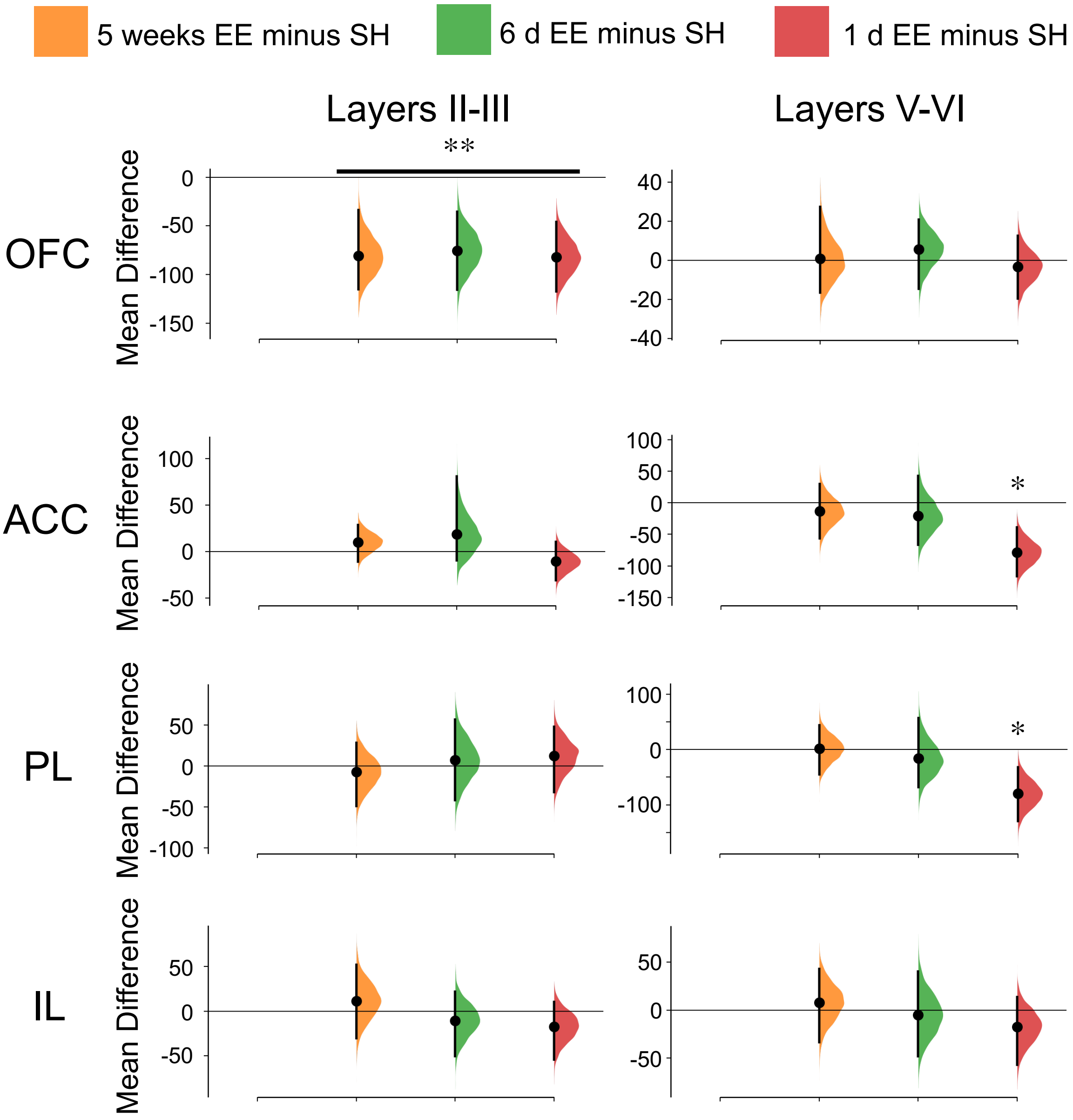

Supporting Figure 3

Laminar-based analyses of Fos expression in prefrontal cortex subareas. The mean difference for 3 comparisons of Fos counts (cells/mm^2) against the shared Standard Housing (SH) condition are shown in the above plots where mean differences are plotted as bootstrap sampling distributions. Each mean difference is depicted as a dot. Each 95% confidence interval is indicated by the ends of the vertical error bars. **p<0.05, **p<0.01* compared to SH. *Legend*: II-III=shallow layers II-III; V-VI=deep layers V-VI
